# Does #Tamojunto alter the dynamic between drug use and school violence among youth? Secondary analysis from a large cluster-randomized trial

**DOI:** 10.1007/s00787-021-01863-x

**Published:** 2021-08-20

**Authors:** Hugo Cogo-Moreira, Julia D. Gusmões, Juliana Y. Valente, Michael Eid, Zila M. Sanchez

**Affiliations:** 1 Faculty of Teacher Education and Languages, Østfold University College, 1757 Halden, Norway; 2 Department of Preventive Medicine, Universidade Federal de São Paulo, Brazil. Rua Botucatu, 740, 4° Andar, São PauloSão Paulo, SP Brazil; 3 Department of Education and Psychology, Methods and Evaluation Division, Freie Universität Berlin, Habelschwerdter Allee 45, Room JK 27/213, Berlin, Germany

**Keywords:** Longitudinal analysis, Drug use, Violence, Cross-lagged model

## Abstract

The present study investigated how intervention might alter the relationship between perpetrating violence and later drug use. A cluster-randomized controlled trial design involving 72 schools (38 intervention, 34 control) and 6390 students attending grades 7 and 8 was employed in Brazil. Drug use and violence were assessed at three points. A random-intercept cross-lagged panel model examined the reciprocal association between drug use and school violence domains across the three data collection waves. For both groups, we found that the cross-lagged effect of perpetration on further drug use in adolescents was stronger than the reverse, but the interrelationship was not statistically significant between #Tamojunto and control schools. The carry-over effects of drug use and violence were also not significantly different between groups. There is a lack of evidence showing that #Tamojunto can modify the dynamics between drug use and school violence across the 21-month period. The direction of the causal effect (i.e., the more perpetration behavior, the more subsequent drug use behavior) is present, but weak in both groups. The trial registration protocol at the national Brazilian Register of Clinical Trials (REBEC) is #RBR-4mnv5g.

**Supplementary Information:**

The online version contains supplementary material available at 10.1007/s00787-021-01863-x.

## Introduction

Violence and drug use appear to have the same etiologies [1] and mechanisms of occurrence [2], tending to co-occur among adolescents [3–5], which makes it difficult to disentangle which comes first. For example, higher rates of violence perpetration and suffering victimization have been associated with increased drug use [6, 7], while increases in drug use have been associated with aggressive behavior [8].

Disentangling the dynamic between two behaviors as complex and overlapping as drug use and violence might be a fundamental step toward understanding how interventions might function, explaining not only if a given intervention might reduce both behaviors concomitantly but also evaluating if the intervention might change the dynamic relationship between the two behaviors.

Statistically, the interplay between drug use and violence can be evaluated via a cross-lagged path model (CLPM), also known as *the cross-lagged regression model*, which examines the reciprocal effects of two or more variables over time using cross-lagged parameters [9]. Cross-lagged models have recently been used in research on drug use and violence; for example, Link and Hamilton [10] used data on male adult offenders to examine short-term changes in substance use and crime over time among a large sample of high-risk former prisoners. They showed that substance use marginally predicted increased odds of re-arrest in one data collection wave, and re-arrest significantly predicted increased odds of substance use in another wave. Among the young, Scholes-Balog et al. [11] used the International Youth Development Study data to longitudinally examine 849 adolescents (53.8% girls) over a five-year period, from grade 7 until 11 in secondary school. The results showed that alcohol use during early and mid-adolescence predicted violence two years later, and further, a bi-directional relationship between adolescent heavy episodic drinking and violence was observed; however, this relationship was not significant when covariates such as family conflict were taken into account.

Methodologically, the studies cited above have some limitations. Although both included three waves of data assessment across time, which allows for robust inference in terms of the interplay between the two behaviors, the authors did not account for the trait and occasion-specific effects of drug use and violence. Dissociation between traits (between-subjects effect) and occasion-specific effects (within-subjects effect) is fundamental to better understanding the process and clearly estimating the causal directionality of the effects [12].

A recent cohort study examined reciprocal associations between drunkenness, drug use, and delinquency at baseline (age 13, grade 7, *N* = 1409, and followed up at grades 8 and 9) adjusting for trait and occasion-specific effects via new advances in the area of structural equation modeling [13], which allowed the incorporation of CLPM random intercepts, creating what is termed the “random intercepts cross-lagged panel model” (RI-CLPM), which accounts for trait-like time-invariant stability [12]. The authors found that delinquency is associated with later drug use or drinking problems, but this relationship was found to be weak.

Can intervention change the interplay between drug use and violence by modifying the dynamics between the two behaviors? To answer this question, two methodological components are necessary: a randomized trial design and a flexible analytical approach to deal with both directionalities (i.e., from drug use to violence and *vice-versa*). Based on Turner et al. [13], we aimed to evaluate if an intervention might change the interplay between both types of behaviors (drug use and violence), testing the effectiveness of #Tamojunto in breaking the directionality of the previously reported effects.

Unplugged, a program developed by the European Drug Addiction Prevention (EU-Dap) Centre, has displayed effectiveness in reducing episodes of drunkenness and frequent marijuana [14] and tobacco and drug use [15] among European adolescents. Based on the “Comprehensive Social Influence Model,” which included among other components “…[the] training of skills to resist the pressure to use drugs, reinforcement of personal attitudes and self-commitments to remain a non-user” (see [16], p. 169), Unplugged has been developed as a standardized package that has been implemented and evaluated in many different languages [16].

The Comprehensive Social Influence Model contains skills training (e.g., communication skills, assertiveness), provides instruction in decision-making, and covers public commitment components differently from narrow-focused social influence programs, which are based on instruction of refusal assertion training and combating direct social influences [17]. In Brazil, it was called *#Tamojunto* and its use has shown a reduction over time in suffering from or practicing bullying and physical violence^1^ and, more recently, binge drinking [18].

## Methods

### Study design

The study was a two-arm cluster-randomized controlled trial, which was conducted among adolescents in grades 7 and 8 attending public school in six Brazilian cities. The aim was to evaluate the #Tamojunto school drug prevention program and compare the results of the integration of the prevention program into school curricula, which would be the intervention condition, with the usual Brazilian curriculum (i.e., no prevention program). The #Tamojunto program is a culturally adapted version of the European prevention program Unplugged [19]. Whereas the intervention has been evaluated by Valente et al. [20], the data have so far not been analyzed with respect to the interplay between violence and drug use over time.

### Sample size and randomization

This study used data from Valente et al. [20], a randomized trial with a cluster structure (children nested in classrooms, and classrooms nested in 72 schools), where the same adolescents were tested across three time points: at baseline (prior to implementing the intervention), a nine-month follow-up, and a 21-month follow-up. Detailed information on the sample size and randomization can be accessed in Valente et al. [20]. Briefly, randomization occurred at the school level using Excel macro [command RAND].

### Measures

The instrument used for data collection was an anonymous questionnaire, developed, tested, and implemented by the EU-Dap team in previous studies [14]. For the Brazilian studies, an adapted and translated version was used [21]. The questionnaires were completed by the participants at the three time points and administered in the classroom by the researchers; teachers were not present in the classrooms during that time.

### Drug use

The questionnaire consisted of five binary responses (“yes” or “no”) on the use of licit or illicit drugs (alcohol, tobacco, inhalants, marijuana, and cocaine) in the past month and a sixth item covering binge drinking in the past month (i.e., the consumption of five or more doses of alcohol during a two-hour period). The score for drug use was obtained by summing the number of “yes” responses (ranging from 0 to 6).

### School violence

Regarding school violence outcomes (termed “violence” throughout the manuscript), the questionnaire assessed two domains: experiencing and perpetrating violence. There were four dichotomous items for each domain (described below) assessing bullying and verbal, physical, and sexual violence in the past month using eight items. “In the past 30 days, have you been verbally/physically/sexually assaulted at your school?” and “In the past 30 days, have you verbally/physically/sexually assaulted anyone at your school?” (“yes” or “no”) [22]. The two items used to assess bullying were “In the past 30 days, how often have your classmates scolded you, bullied you, or teased you so much that you were hurt, harassed, annoyed, offended, or humiliated?” and “In the past 30 days, have you scolded, mocked, manipulated, intimidated, or teased any of your classmates so much that they were hurt, annoyed, offended, or humiliated?” For the analysis, two scores were obtained: one for victimization where the victimization items were summed (score ranging from 0 to 4) and the same procedure to create the perpetration score (also ranging from 0 to 4).

This process of summing is referred as *parceling* and is based on the Aggregation Principle [23–25] and on the Law of Large Numbers [23, 26]. A given parcel will have a larger proportion of true-score variance to unique variance than any item used to build it [27]. As a consequence, the higher the number of items summed, the higher the proportion of true-score variance [26]. Evidence regarding the longitudinal psychometric invariance for the school violence items is described in Supplementary Material 1.

### Socio-demographics

Age, gender, and socioeconomic status (SES) were also assessed. SES measured via the Brazilian Association of Research Companies scale (ABEP) [28], which takes into consideration the head-of-household’s educational level as well as the goods and services used. The scores range from 0 to 46, where higher scores indicate higher SES. This is a tool extensively used in Brazilian studies [29–31] that provides information regarding socioeconomic stratification; details regarding its validation can be accessed in Kamamura and Mazzon [32].

### Statistical analysis

#### The model

To answer whether #Tamojunto can change the interplay between drug use and violence by modifying the dynamics between the two behaviors, we used two multi-group RI-CLPMs [12]: one was to test the effects of the intervention on (a) drug use and victimization and the other on (b) drug use and perpetration. To evaluate the directionality of the effects (from drug use to violence or vice-versa), we analyzed the cross-lagged effects. These were estimated by regressing an occasion-specific drug-use variable on the occasion-specific violence variable one occasion before and vice versa. To evaluate carry-over effects (inertia), we evaluated the autoregressive effects by regressing the occasion-specific variable on its previous occasion-specific latent variable (i.e., drug nine months on drug baseline).

**Fig. 1:**
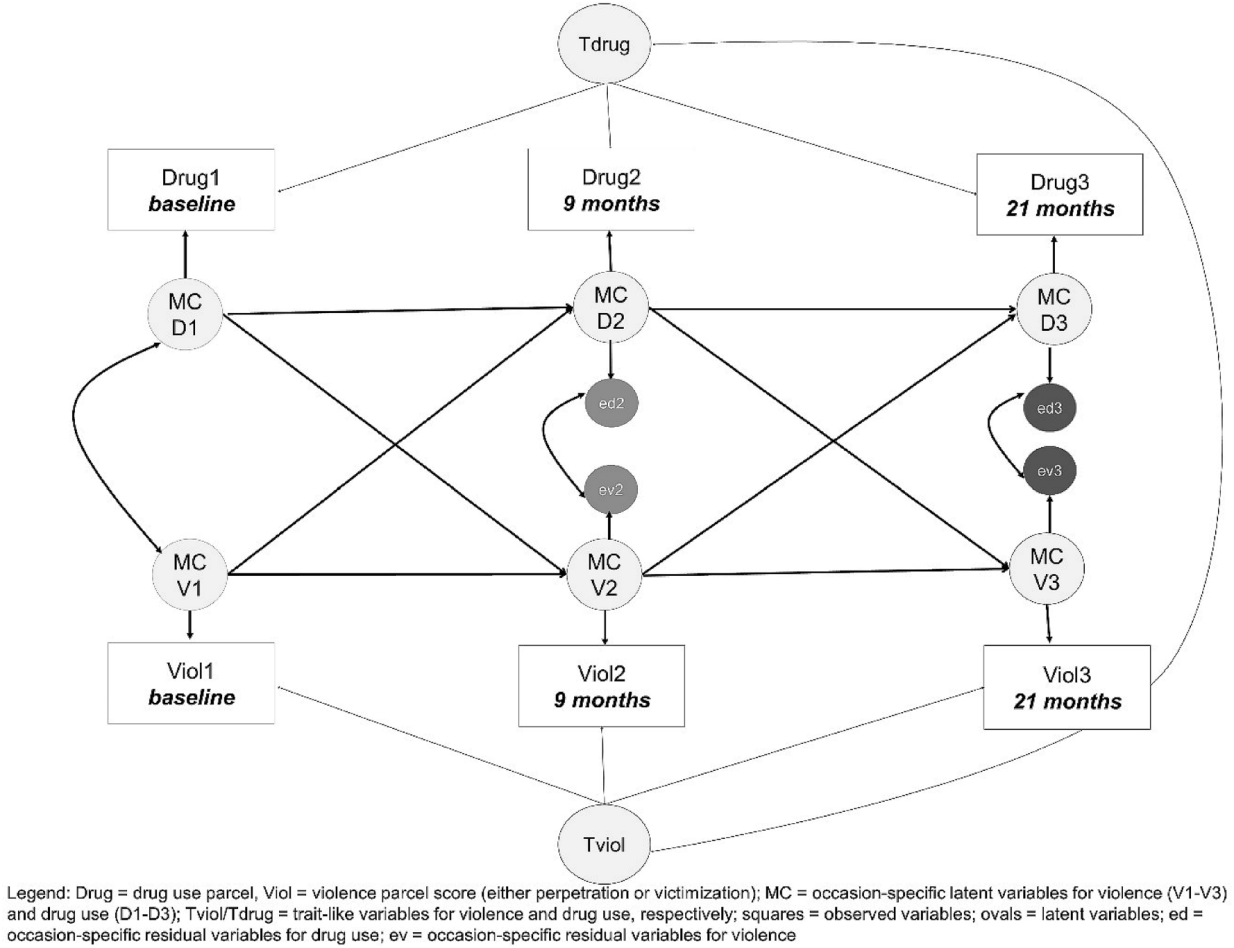
RI-CLPM model

To make sure that both above-described parameters (i.e., cross-lagged and autoregressive) characterized the process on the within-subjects level (intra-individual behavior over time), we included a latent trait variable accounting for stable inter-individual differences over time. We have for each RI-CLPM model two traits: (a) drug use trait and victimization trait; and (b) drug use trait and perpetration trait. These trait-like individual difference variables and are allowed to be correlated, showing how both constructs are associated at between subject levels. As a consequence of the definition of these trait variables, the process can be modeled on the level of occasion-specific variables that characterize occasion-specific deviations from the general trait level that are due to situational influences and/or the interaction between the individual and the situation, but also due to measurement error. The general structure of the model with all correlations allowed is depicted in Fig. 1. The syntaxes for analyzing this model with the computer programs Mplus and Lavaan are available at https://ellenhamaker.github.io/RI-CLPM/. Mulder and Hamaker [33] provided syntaxes for what they called *basic* multi-group RI-CLPM, in which no restrictions were added to the within effects, and a more restricted multi-group version of RI-CLPM, in which cross-lagged parameters (i.e., cross-lagged and autoregressive effects) were fixed to be equal between groups to ensure that the dynamic processes were the same. This procedure allows to evaluate if the dynamics on the within-subject level is equal across groups.

#### Estimator, multilevel data, and dealing with missing data

The maximum likelihood robust (MLR) estimator was applied in all analyses, which uses the Huber–White Sandwich estimator to estimate robust standard errors dealing with the multilevel design (i.e., chidlren nested in schools) as described by Asparouhov [34, 35] and with missingness across time by assuming missing-at-random (MAR) mechanism invoking full-information maximum likelihood, which is an efficient method for handling missing data [36]. We are assuming missing-at-random that the missingness depends on drug use and violence, which are variables in the longitudinal model. In other words, if the future missingness only depends on earlier drug use and violence, the MAR assumption is fulfilled by having these variables in the model.

### Model evaluation

The following fit indices and their respective cut-offs were used to evaluate the RI-CPLM’s goodness of fit as suggested by Schermelleh-Engel et al. [37]: comparative fit index (CFI), root mean square error of approximation (RMSEA), standardized root mean square residual (SRMR), and χ^2^ *p*-value. An RMSEA value equal to or smaller than 0.05 indicates a good approximate model fit. The *p* value of the corresponding test of approximate fit should be equal to or less than 0.05. The CFI value should be greater than or equal to 0.97. The hypotheses of perfect fit can be tested by a χ^2^ test and the corresponding *p *value should be equal to or smaller than 0.05. The Satorra–Bentler chi-square difference test under robust maximum likelihood [38] was used to judge whether the restrictions (equality of cross-lagged effects between #Tamojunto and control group) imposed to the *basic* multi-group RI-CLPM model did not worsen the basic model fit.

All models were run using Mplus version 8.5 [39] and the syntaxes are available upon request.

## Results

Table 1 presents the descriptive statistics for the observed drug use and two school violence variables, as well as the correlations and co-variances of the variables over time. The main socio-demographic features of the students, per group, were as follows: the control group’s mean age was 12.59 years (standard deviation [SD] of 0.82), 51.43% were female, and the mean ABEP score was 27.9 (SD = 8.16), which corresponds to a medium–low SES. For the intervention group, the average age was 12.64 years (SD = 0.83), 50.68% were female, and the mean ABEP score was 28.12 (SD = 8.17). Further details about school-level features can be found in Supplementary Table 1 in Valente et al. [20].

**Table 1:** Descriptive statistics for the nine outcomes across the three waves per group

Outcomes	Sample size	Mean	Variance	Control		%Min	%Max	Sample size	Mean	Variance	Intervention		%Min	%Max
				Minimum	Maximum						Minimum	Maximum		
Drug (Baseline)	3081	0.331	0.632	0	5	82.77	0.26	3157	0.351	0.692	0	6	82.26	0.1
Drug at 9 months	2146	0.384	0.756	0	6	80.2	0.09	1995	0.408	0.791	0	5	79.4	0.25
Drug at 21 months	1839	0.558	1.025	0	6	71.56	0.22	1749	0.599	1.1	0	5	70.27	0.51
Perpetration (Baseline)	3035	0.378	0.582	0	4	74.73	0.89	3131	0.417	0.682	0	4	73.75	1.18
Perpetration at 9 months	2133	0.456	0.646	0	4	69.76	0.42	1969	0.492	0.715	0	4	68.92	0.41
Perpetration at 21 months	1817	0.477	0.673	0	4	68.68	0.55	1743	0.523	0.778	0	4	67.53	0.63
Victimization (Baseline)	3000	0.496	0.639	0	4	66.07	0.17	3113	0.58	0.773	0	4	61.84	0.45
Victimization at 9 months	2115	0.601	0.701	0	4	59.15	0.09	1960	0.658	0.805	0	4	57.65	0.2
Victimization at 21 months	1800	0.558	0.663	0	4	61.56	0.11	1732	0.618	0.759	0	4	59.41	0.17

Table 1 shows that the mean scores for drug use increased across time for both groups and that there was an inflation at zero (that is, a floor effect, with many responses of zero) across the nine observed parcels. Although the missing values increased across the data collection waves (see the flowchart in Supplementary Material 2), as would be expected for any longitudinal design, the covariance coverage (proportion of data present) was, in every scenario, never less than 40% of the data available, which is more than enough to compute a trustworthy covariance matrix (see Supplementary Material 3 for the covariance coverage). The number of analyzed cases, complying with the intention-to-treat paradigm, was 6,390 (control n = 3148 and #Tamojunto n = 3,242).

**Fig. 2:**
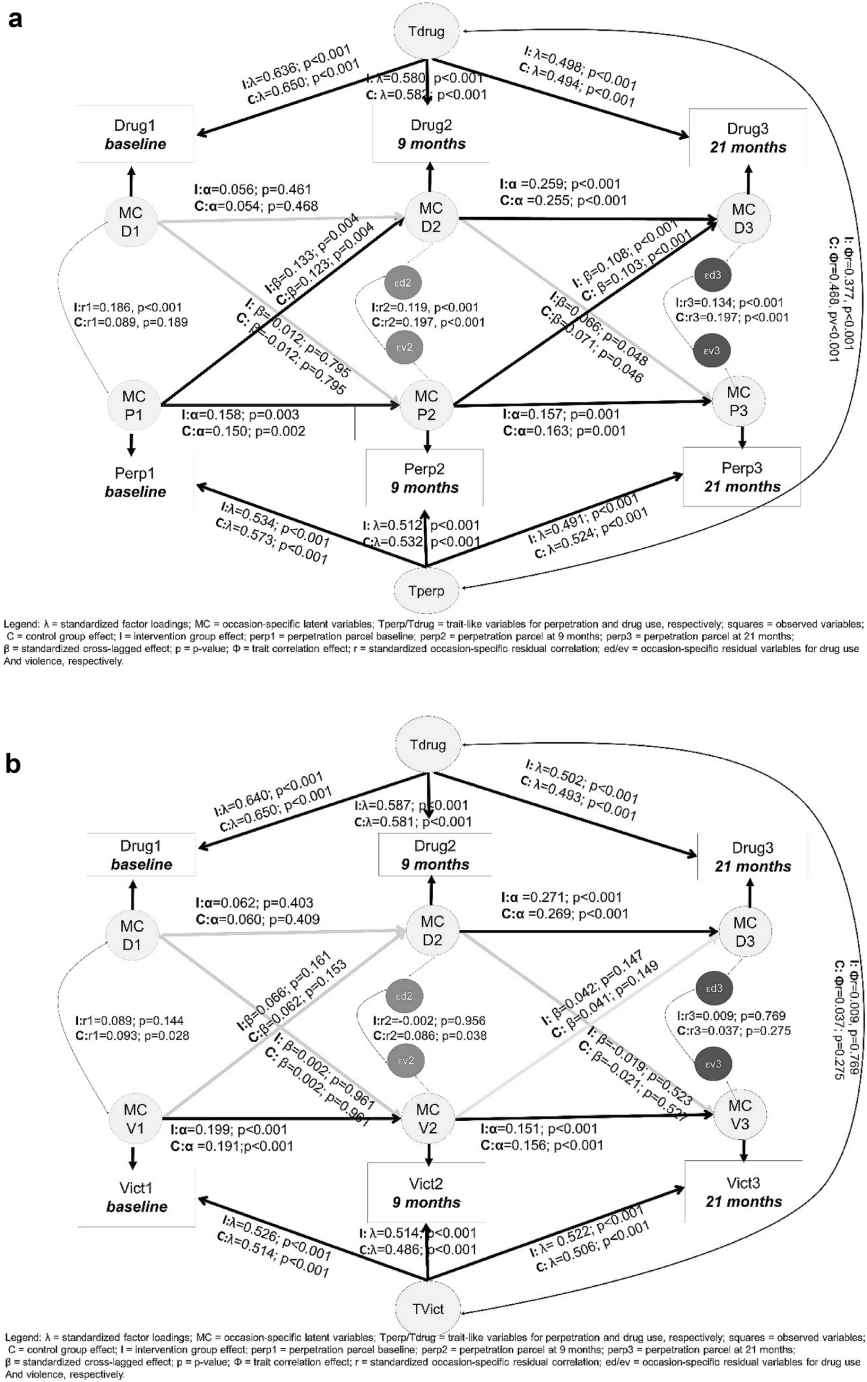
**a** RI-CLPM under restrictive specification for drug use and perpetration. **b** RI-CLPM under restrictive specification for drug use and victimization

Figure 2a, b shows the diagram of the RI-CLPM with standardized effects for both restricted models for perpetration and victimization, respectively.

For both groups, the cross-lagged effects (i.e., within-subjects effects) from perpetration to drugs were stronger (i.e., standardized cross-lagged parameter estimates ranging from 0.103 to 0.133) compared to the cross-lagged effects from drugs to perpetration (i.e., ranging from 0.012 to 0.71) (see Fig. 2). For the victimization model, the cross-lagged effects were not statistically significant and had all standardized effects below 0.066. Drug use auto-regressive effects increased over time, and they were significantly different from 0 only for the last lag (i.e., from 9 to 21 months). Moreover, for the last lag, autoregressive effects were stronger for drug use (ranging from 0.255 to 0.271) than for school violence (ranging from 0.157 to 0.163 for perpetration, and from 0.151 to 0.156 for victimization).

Between-persons associations (i.e., correlation between the traits) suggested that, on average, drug use and perpetration were positively associated over time and the effect size of this correlation was moderate (r_control_ = 0.377, *P* < 0.01 and r_intervention_ = 0.468, *P* < 0.01). For victimization, the trait correlations were close to 0.

Table 2 shows that the restricted models (i.e., where cross-lagged and autoregressive effects were constrained to be equal between both the #Tamojunto and control groups) for a) the interplay between drug use and victimization and b) the interplay between drug use and perpetration showed good fit indices. Moreover, the Satorra–Bentler chi-square difference test comparing the constrained models against less restrictive models (i.e., where the cross-lagged and autoregressive effects are free to be different between the groups) was not statistically significant, meaning that adding constraints did not worsen model fit. Hence, there is no evidence that the within-subjects effects differ between the two groups for drug use and victimization interplaying (Satorrra–Bentler χ^2^ difference test P = 0.179) or drug use and perpetration interplaying (Satorrra–Bentler χ^2^ difference test P = 0.163). In other words, we lack evidence that #Tamojunto can change the interplay between drug use and violence.

**Table 2:** Fit indices for basic and restrictive models

Model fit indices	Drug × Victimization		Drug × Perpetration	
	Basic Model	Constraint Model	Basic Model	Constraint Model
Chi-square test of model fit	0.241	12.249	19.101	25.287
Degree of freedom	2	10	2	10
*p* value	0.8864	0.2687	0.0001	0.0048
Scaling correction	1.0562	1.3654	0.9216	1.3917
RMSEA	0	0.008	0.052	0.022
90% Confidence Interval of RMSEA	0 to 0.017	0 to 0.022	0.032 to 0.074	0.011 to 0.033
RMSEA Close fit	1	1	0.4	1
Comparative fit index	1	0.999	0.991	0.992
Satorrra–Bentler Chi-square difference test		0.1792		0.1673

## Discussion

This study was conducted using data from a wider cluster-randomized trial to assess the effect of #Tamojunto on drug use prevention [20], where three different patterns of drug use behavior (abstainers/low users, alcohol users/binge drinkers, and polydrug users) where identified across the waves of evaluation. The primary outcome was focused on drug use and showed that #tamojunto was not successful in changing adolescent drug use patterns over time. Given the complexity of drug use and school violence behaviors and how they overlap, it is difficult to understand which one occurs first and whether one is more influential than the other. Hence, this manuscript brings the interplaying between both behaviors in the context of a RCT.

Turner et al. [13] showed that delinquency is associated with later drug use or drinking problems, also previously reported by others but not using RI-CLPM. Here, we intended to go beyond asking whether intervention can change such a dynamic. Randomized trials with more than two waves of assessment might clarify these issues. A search of the existing literature suggests that this is the first study analyzing the effects of Unplugged on the dynamic of drug use and school violence. We found a lack of evidence for any difference in dynamics between the groups after the implementation of the program. However, perpetrating violence was a predictor of future increased drug use for both groups, and although the magnitude of the effect is small, it matches that described previously by Turner et al. [13] indicating the robustness and replicability of the present findings.

RI-CLPM simultaneously integrates two requisites for establishing causal relations, namely establishing an association between the variables studied and taking into account the time order of the processes (i.e., the cause has to occur before the result) [40]. Therefore, the analyses represent an important step forward as most of the previous studies on this topic with a longitudinal design assumed a pre-specified antecedent and consequent to be tested: either drug use on violence [4, 41] or violence on drug use [42, 43]. In terms of testing the impact of #Tamojunto on the dynamics, this is the first study where drug use and violence have been assessed concomitantly.

Compared with Turner et al.’s recent RI-CLPM [13], this study did not find large between-subjects effects—the correlation between drug use and violence across the 21 months ranged from 0.37 to 0.47 and only between drug use and perpetration of violence (but not victimization of violence).". Turner et al. [13] found moderate and high correlations of the trait behavior (i.e., between-subjects effects) of delinquency and drug use (r = 0.6, *P* < 0.001) and delinquency and drunkenness (r = 0.93, *P* < 0.001). Furthermore, in terms of cross-lagged paths, delinquency in Turner et al. [13] was associated with later drug use across grades 7 to 9, but drug use was not associated with later delinquency or drunkenness at any time point. Therefore, these latter results converge with our (also weak) results at the individual level, where weak (ranging from β = 0.093 to 0.111) systematic significance effect sizes stemming from violence and posterior drug use were found.

Epidemiologically, our findings provide support for the theoretical model that proposes that early violent behavior has a direct effect on drug use. One possible explanation for this may be that adolescents may have engaged in substance use as a way of coping with stressful life circumstances; previous studies have shown that perpetrating and experiencing violence are linked with adjustment problems [44]. In addition, adolescents who engage in violent behaviors increasingly spend time with peers who are engaging in multiple forms of risk, including substance use [45].

In terms of the auto-regressive effects, the obtained findings are similar to those previously described where early episodes of violence predict later episodes of violence, as well as the fact that earlier drug use predicts more use later in time [4, 46–48]. However, it is important to note that the carry-over effects of drug use were stronger than those of violence.

In terms of the carry-over effects of violence, research has shown that personal traits of aggressiveness, impulsiveness, and consequent lack of self-control increase the person’s involvement in violent acts later in life [49]. It is also true that those who experience violent victimization tend to continue suffering from it [50].

We raised two hypotheses regarding the fact that the intervention did not change the dynamic of perpetrating violence–drug use. The first one is related to the nature of the outcomes being assessed, where a robust amount of the variance is related to trait, which is an enduring feature, leaving a small room for cross-lagged effects being robust in between group difference. The second one is related to the intervention’s impact per se*,* which was not powerful enough to modify the main outcomes related to drug as described by [20]. It is important to note, however, that a positive effect on one of the outcomes under evaluation is not a prerequisite for applying a RI-CLPM. The RI-CLPM allows a more detailed analysis of intervention effects by comparing different aspects of stability and change across intervention and control groups in a sophisticated way.

In conclusion, this study adds to the existing literature by suggesting that early violent behavior predicts further drug use among adolescents and shows lack of evidence for #Tamojunto changing the dynamics between drug use and school violence (perpetration and victimization). Preventive programs should target not only drug use but also perpetration behaviors given that they are a predictor of increased drug use.

## Supplementary Information

Below is the link to the electronic supplementary material.

Supplementary file1 (DOCX 14 kb)

Supplementary file2 (DOCX 45 kb)

Supplementary file3 (DOCX 15 kb)
