# Supplementary material for: Does #Tamojunto alter the dynamic between drug use and school violence among youth? Secondary analysis from a large cluster-randomized trial
Source: Eur Child Adolesc Psychiatry. 2021 Aug 20;32(2):293–302. doi: 10.1007/s00787-021-01863-x (PMC9971055; doi:10.1007/s00787-021-01863-x)
Supplement: Supplementary file 1 — Supplementary file1 (DOCX 14 kb) [file 787_2021_1863_MOESM1_ESM.docx]

Supplementary material 1 – Longitudinal invariance of the violence scale at item-level measurement

Due to lack of evidence regarding invariance for the questionnaire to assess school violence, the following hierarchically nested model for such scales was specified: configural, metric, strong, and strict invariance. Due to our robust sample size (n > 6,000), we used the cutoff proposed by Meade, Johnson and Braddy (35) test if the invariance holds where changes in CFI values are less than or equal to 0.002, or a ΔCFI <= 0.002 indicates that the more constrained model should not be rejected.

Supplementary table 1 – Longitudinal invariance of the violence scale with eight dichotomous items

| Features of the Model/fit indices | Configural | Weak | Strong | Strict |
| --- | --- | --- | --- | --- |
| Free parameters | 51 | 37 | 21 | 19 |
| χ² | 1235.678 | 1279.149 | 1328.816 | 1337.085 |
| Degrees of Freedom | 249 | 263 | 279 | 281 |
| P-value | <0.0001 | <0.0001 | <0.0001 | <0.0001 |
| RMSEA | 0.025 | 0.025 | 0.024 | 0.024 |
| 90% RMSEA | 0.024 to 0.026 | 0.023 to 0.026 | 0.023 to 0.026 | 0.023 to 0.026 |
| Close fit | 1 | 1 | 1 | 1 |
| CFI | 0.934 | 0.932 | 0.930 | 0.929 |
| TLI | 0.927 | 0.928 | 0.930 | 0.930 |

Supplementary table 1 showed presents the psychometric features of the violence items across the three time points. We were able to achieve scalar invariance (i.e. factor loading and thresholds were held equal across the three time points). Constraints on factor loadings and thresholds did not worsen the scalar model when compared to the metric model (ΔCFI=0.002), where only factor loadings were held equal across the three time points. This revealed evidence of the invariance of the fitted unidimensional violence behavior across the three waves. Based on that, we felt comfortable proceeding with the parceling procedures involving the violence scale.
