# Supplementary material for: Does #Tamojunto alter the dynamic between drug use and school violence among youth? Secondary analysis from a large cluster-randomized trial
Source: Eur Child Adolesc Psychiatry. 2021 Aug 20;32(2):293–302. doi: 10.1007/s00787-021-01863-x (PMC9971055; doi:10.1007/s00787-021-01863-x)
Supplement: Supplementary file 2 — Supplementary file2 (DOCX 45 kb) [file 787_2021_1863_MOESM2_ESM.docx]

**Supplementary Material 2:** CONSORT flow diagram for the cluster randomized controlled trial

**Assessed**

Schools n = 72

Classes n = 261

**Control arm**

Schools n = 34

Classes n = 131

**Intervention arm**

Schools n = 38

Classes n = 130

**Baseline**

**(February/2014)**

Schools n = 34

Classes n = 131

Absent n = 752

Refusal n = 40

Respondents n = 3,318

Valid n = 3,144

**Baseline**

**(February/2014)**

Schools n = 38

Classes n = 130

Absent n = 778

Refusal n = 19

Respondents n = 3,340

Valid n = 3,239

**9-month follow-up**

**(November/2014)**

Schools n = 34

Classes n = 131

Absent n = 1,008

Refusal n = 31

Respondent n = 3,044

Valid n = 2,189

**9-month follow-up**

**(November/2014)**

Schools n = 38

Classes n = 130

Absent n = 1,119

Refusal n = 21

Respondent n = 2,913

Valid n = 2,042

**21-month Follow-up**

**(November/2015)**

Schools n = 33

Classes n = 128

Absent n = 966

Refusals n = 20

Respondent n = 2279

Valid n = 1,861

**21-month Follow-up**

**(November/2015)**

Schools n = 37

Classes n = 128

Absent n = 1.180

Refusals n = 37

Respondent n = 2155

Valid n = 1,774

**Analysed**

3143

**Analysed**

3243

Absent= absent from school in moment of the assessment

Refusals= subjects who refusals to participate of the assessment

Valid= number of subjects used in the cross-sectional analysis

Respondent= participants assenting to participate, providing data
