# Supplementary material for: Does #Tamojunto alter the dynamic between drug use and school violence among youth? Secondary analysis from a large cluster-randomized trial
Source: Eur Child Adolesc Psychiatry. 2021 Aug 20;32(2):293–302. doi: 10.1007/s00787-021-01863-x (PMC9971055; doi:10.1007/s00787-021-01863-x)
Supplement: Supplementary file 3 — Supplementary file3 (DOCX 15 kb) [file 787_2021_1863_MOESM3_ESM.docx]

Supplementary Material 3 – Covariance coverage per group

|  |  | Drug1 | Drug2 | Drug3 | Vict1 | Vict2 | Vict3 |
| --- | --- | --- | --- | --- | --- | --- | --- |
|  | DRUG1 | 0.979 |  |  |  |  |  |
|  | DRUG2 | 0.663 | 0.682 |  |  |  |  |
| Control Group | DRUG3 | 0.569 | 0.464 | 0.584 |  |  |  |
|  | VIOL1 | 0.937 | 0.642 | 0.556 | 0.953 |  |  |
|  | VIOL2 | 0.653 | 0.659 | 0.458 | 0.633 | 0.672 |  |
|  | VIOL3 | 0.558 | 0.455 | 0.566 | 0.545 | 0.449 | 0.572 |
|  |  |  |  |  |  |  |  |
|  |  | Drug1 | Drug2 | Drug3 | Vict1 | Vict2 | Vict3 |
|  | DRUG1 | 0.974 |  |  |  |  |  |
|  | DRUG2 | 0.597 | 0.615 |  |  |  |  |
| #Tamojunto Group | DRUG3 | 0.523 | 0.402 | 0.539 |  |  |  |
|  | VIOL1 | 0.943 | 0.583 | 0.515 | 0.960 |  |  |
|  | VIOL2 | 0.587 | 0.593 | 0.395 | 0.574 | 0.605 |  |
|  | VIOL3 | 0.518 | 0.399 | 0.527 | 0.511 | 0.379 | 0.393 |

Legend: drug = drug use measures; vict = victimization scores

|  |  | Drug1 | Drug2 | Drug3 | Perp1 | Perp2 | Perp3 |
| --- | --- | --- | --- | --- | --- | --- | --- |
|  | DRUG1 | 0.979 |  |  |  |  |  |
|  | DRUG2 | 0.663 | 0.682 |  |  |  |  |
| Control Group | DRUG3 | 0.569 | 0.464 | 0.584 |  |  |  |
|  | VIOL1 | 0.947 | 0.652 | 0.560 | 0.964 |  |  |
|  | VIOL2 | 0.659 | 0.665 | 0.452 | 0.648 | 0.678 |  |
|  | VIOL3 | 0.563 | 0.461 | 0.572 | 0.553 | 0.458 | 0.577 |
|  |  |  |  |  |  |  |  |
|  |  | Drug1 | Drug2 | Drug3 | Perp1 | Perp2 | Perp3 |
|  | DRUG1 | 0.974 |  |  |  |  |  |
|  | DRUG2 | 0.597 | 0.615 |  |  |  |  |
| #Tamojunto Group | DRUG3 | 0.523 | 0.402 | 0.539 |  |  |  |
|  | VIOL1 | 0.945 | 0.590 | 0.518 | 0.966 |  |  |
|  | VIOL2 | 0.589 | 0.596 | 0.397 | 0.583 | 0.607 |  |
|  | VIOL3 | 0.521 | 0.401 | 0.530 | 0.518 | 0.396 | 0.538 |

Legend: drug = drug use measures; perp = perpetration scores
